# Autophagic lysosome reformation dysfunction in glucocerebrosidase deficient cells: relevance to Parkinson disease

**DOI:** 10.1093/hmg/ddw185

**Published:** 2016-07-04

**Authors:** Joana Magalhaes, Matthew E. Gegg, Anna Migdalska-Richards, Mary K. Doherty, Phillip D. Whitfield, Anthony H.V. Schapira

**Affiliations:** 1 Department of Clinical Neurosciences, Institute of Neurology, University College London, Rowland Hill Street, London, UK; 2 Lipidomics Research Facility, University of the Highlands and Islands, Inverness, UK

## Abstract

Glucocerebrosidase *(GBA1*) gene mutations increase the risk of Parkinson disease (PD). While the cellular mechanisms associating *GBA1* mutations and PD are unknown, loss of the glucocerebrosidase enzyme (GCase) activity, inhibition of autophagy and increased α-synuclein levels have been implicated. Here we show that autophagy lysosomal reformation (ALR) is compromised in cells lacking functional GCase. ALR is a cellular process controlled by mTOR which regenerates functional lysosomes from autolysosomes formed during macroautophagy. A decrease in phopho-S6K levels, a marker of mTOR activity, was observed in models of GCase deficiency, including primary mouse neurons and the PD patient derived fibroblasts with *GBA1* mutations, suggesting that ALR is compromised. Importantly Rab7, a GTPase crucial for endosome-lysosome trafficking and ALR, accumulated in GCase deficient cells, supporting the notion that lysosomal recycling is impaired. Recombinant GCase treatment reversed ALR inhibition and lysosomal dysfunction. Moreover, ALR dysfunction was accompanied by impairment of macroautophagy and chaperone-mediated autophagy, increased levels of total and phosphorylated (S129) monomeric α-synuclein, evidence of amyloid oligomers and increased α-synuclein release. Concurrently, we found increased cholesterol and altered glucosylceramide homeostasis which could compromise ALR. We propose that GCase deficiency in PD inhibits lysosomal recycling. Consequently neurons are unable to maintain the pool of mature and functional lysosomes required for the autophagic clearance of α-synuclein, leading to the accumulation and spread of pathogenic α-synuclein species in the brain. Since GCase deficiency and lysosomal dysfunction occur with ageing and sporadic PD pathology, the decrease in lysosomal reformation may be a common feature in PD.

## Introduction

Gaucher disease (GD), the most common lysosomal disease, is caused by homozygous mutations in the *glucocerebrosidase 1 (GBA1)* gene which encodes the lysosomal enzyme glucocerebrosidase (GCase). GCase is responsible for the conversion of glucosylceramide to glucose and ceramide in the lysosome. Deficiency in GCase activity leads to accumulation of its substrate in the lysosome and compromised lysosomal activity (1,2).

The pathology of Parkinson disease (PD) is characterized by loss of substantia nigra pars compacta dopaminergic neurons with the formation of α-synuclein-rich Lewy bodies in surviving neurons (3). A subset of GD patients and *GBA1* mutation carriers are at high risk of developing PD (4–7). Mutations in the *GBA1* gene are numerically the most important genetic risk factor for developing PD (7,8). The patients with PD with *GBA1* mutations have similar clinical features and pathology to sporadic PD (8,9), except there is an earlier age of onset (about 5 years) and increased cognitive decline (8–10). The biochemical link between *GBA1* mutations and PD was established with the identification of GCase deficiency in PD brains positive for these mutations (11). This deficiency was most pronounced in the substantia nigra and other midbrain areas, sites of the greatest pathology in PD. Of particular importance was the demonstration that there was evidence of significant GCase deficiency in sporadic PD brains (11–13) suggesting loss of enzyme activity is central to PD pathogenesis. Loss of GCase activity has been shown to promote α-synuclein accumulation (14–17) and mitochondrial dysfunction (18,19). However, the mechanism by which GCase deficiency leads to α-synuclein accumulation is still unclear.

GCase deficiency and the subsequent accumulation of its substrate affect lipid metabolism and trafficking, decreases lysosomal degradation capacity and impairs autophagy (5). The autophagy – lysosomal pathway (ALP) is important in the clearance of α-synuclein and the impairment of both macroautophagy and chaperone mediated autophagy (CMA) have been linked to PD pathogenesis (20–23).

Since lysosomes are the primary degradative compartment of the cell, their biogenesis and recycling are vital for cellular function. Lysosome biogenesis requires the endosomal system to direct newly synthesized lysosomal proteins to the lysosome. Lysosomal targeted substrates pass through different endosomal intermediaries to reach their final destination. Endosomes mature from early endosomes (Rab5 positive) to late endosomes (Rab7 positive) which then fuse with the lysosome, delivering the cargo. This process is important for delivery of essential proteins for lysosomal function (24). Recently Yu *et al.* (25) described lysosomal reformation following autophagy termination; this pathway is used to recycle autolysosomes in order to maintain the cellular pool of dense and functional lysosomes. After the degradation of the autolysosomal products by autophagy, mTOR is reactivated leading to the attenuation of autophagy and to the formation of proto-lysosomal tubules and vesicles that are ultimately excluded from the autolysosomes and mature into functional lysosomes, in a process dependent of Rab7 (25). This process, termed autophagy lysosome reformation (ALR), was found to be inhibited in fibroblasts derived from patients with the lysosomal storage disorders Scheie syndrome and Fabry disease (25).

The relationship between loss of GCase activity in the lysosome and PD is not yet fully understood. Here we describe for the first time the importance of GCase in lysosomal reformation. We show that GCase deficient cells exhibit altered lysosomal recycling, and propose that the accumulation of defective lysosomes contributes to autophagy impairment and accumulation of α-synuclein.

## Results

### GCase deficiency affects lysosomal recycling in *Gba1* knockout mouse embryonic fibroblasts (MEFs) and in patient derived fibroblasts with *GBA1* mutations

**Figure 1.**
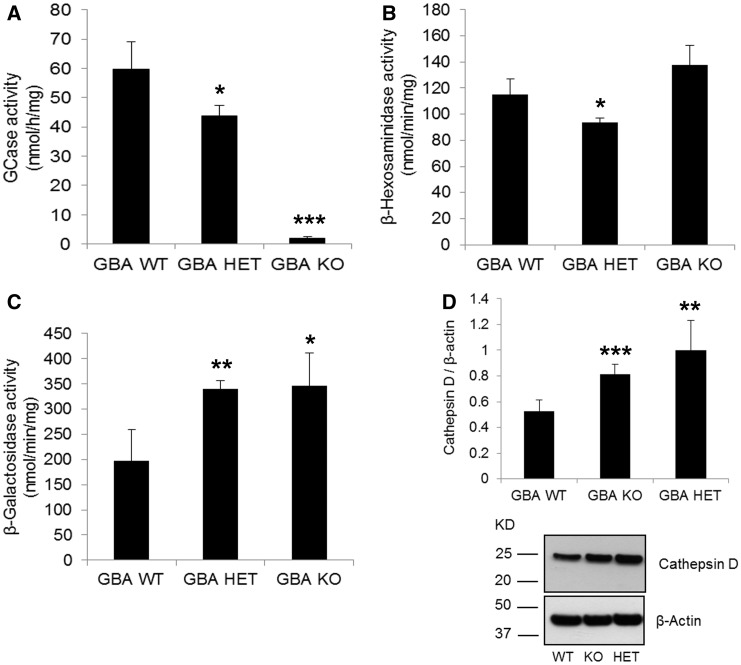
GCase deficiency in *Gba1* HET and *Gba1* KO MEFs causes changes in lysosomal content. (**A**) GCase activity was decreased in *Gba1* HET and in *Gba1* KO compared to *Gba1* WT (*n =* 3). (**B**) β-hexosaminidase activity was decreased in *Gba1* HET but unchanged in *Gba1* KO compared to *Gba1* WT (*n =* 3). (**C**) The activity of β-Galactosidase was significantly increased in *Gba1* HET and *Gba1* KO compared to *Gba1* WT (*n =* 5). (**D**) Mature cathepsin D protein levels (28 kDa) were increased in both *Gba1* KO and *Gba1* HET compared to *Gba1* WT (*n =* 4). All data represent mean ± SD, * *P <* 0.05; ** *P <* 0.01, *** *P <* 0.001.

*Gba1* wild-type (WT), heterozygote (HET) and knockout (KO) MEFs were generated from *Gba1* transgenic mice (26). As expected *Gba1* HET cells had significantly decreased GCase activity (27% decrease, *P <* 0.05), compared to WT, while *Gba1* KO presented minimal activity (3% residual activity) (Figure 1A) . The activities or expression of other lysosomal proteins were also affected. β-hexosaminidase was unaffected in *Gba1* KO but was significantly decreased (20%, *P <* 0.05) in *Gba1* HET cells (Figure 1B). β-galactosidase activity was increased in both *Gba1* HET (72%, *P <* 0.005) and *Gba1* KO cells (75%, *P <* 0.05; Figure 1C). Mature cathepsin D protein levels (∼ 28 kDa) were increased in both *Gba1* HET (88%, *P <* 0.05) and KO (53%, *P <* 0.01) compared to *Gba1* WT cells (Figure 1D). No changes were detected in cathepsin D mRNA levels (*Gba1* WT, 0.067 ± 0.03; *Gba1* KO, 0.060 ± 0.03; *Gba1* HET, 0.049 ± 0.02). LAMP1 protein levels were unaffected in *Gba1* HET and *Gba1* KO MEFs compared to *Gba1* WT (*Gba1* HET 116.3 ± 10.4%, *Gba1* KO 127.2 ± 50.6%). Lipidomic analysis revealed an accumulation of several glucosylceramide species in *Gba1* KO and *Gba1* HET cells (Supplementary Material, Fig. SI 1).

The lysosomal dysfunction detected could be related to the accumulation of dysfunctional lysosomes due to impaired lysosomal recycling via ALR. The recycling process is regulated by activation of mTOR, and is measured via phosphorylation of its substrate p70S6Kinase (phopho-S6K) (25).

**Figure 2.**
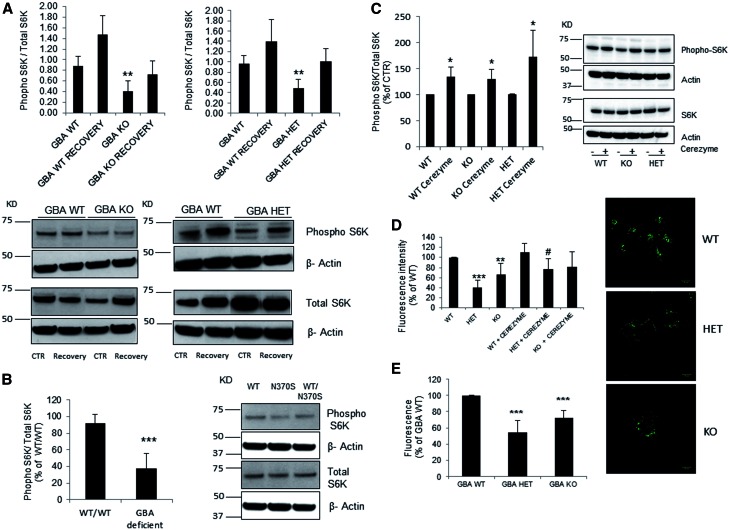
Impairment of autophagic lysosome reformation in GCase deficient cells. (**A**) *Gba1* KO and *Gba1* HET presented decreased basal levels of phopho-S6K compared to *Gba1* WT (*n =* 4). The percentage of recovery was similar between all cell lines (40-50% increase compared with basal) however the levels of phopho-S6K were consistently lower in *Gba1* KO and *Gba1* HET cells. (**B**) Phopho-S6K protein levels were significantly lower in mutant *GBA1* patient derived fibroblasts (*n =* 5) compared to WT fibroblasts. (**C**) Phospho-S6K levels were significantly increased in WT, HET, KO *Gba1* MEF cells upon cerezyme treatment (*n =* 4). Results were expressed as % of respective control. (**D**) Lyso ID fluorescence measurements detected a significant decrease in acidic functional lysosomes in both *Gba1* HET and *Gba1* KO compared to *Gba1* WT (*n =* 4). Cerezyme treatment significantly increased acidic functional lysosomes in *Gba1* HET MEFs but not *Gba1* KO. Results were expressed as % of respective control. ^#^*P <* 0.01 for HET + cerezyme compared to HET. Scale bar 10µm. (**E**) Lyso ID fluorescence measurements using a plate reader detected a significant decrease in functional/acidic lysosomes in both *Gba1* HET and *Gba1* KO compared to *Gba1* WT (*n =* 4). All data represent mean ± SD, *P < 0.05; **P < 0.01, ***P < 0.001 vs. respective control.

We analyzed ALR in the *Gba1* deficient MEFs. Under basal conditions (CTR), *Gba1* KO and *Gba1* HET displayed decreased levels of phopho-S6K compared to *Gba1* WT (respectively 47%, *P <* 0.01, and 50%, *P <* 0.01; Figure 2A). Similar to a previous report (25), starvation for 2 h inhibited mTOR resulting in undetectable levels of phospho-S6K (data not shown). After being fed with fresh complete media and incubated for 1h (recovery), phopho-S6K was detected (Figure 2A), with once again less in GCase deficient cells compared to WT. When cells recovered for an extra hour (2h starvation followed by 2h recovery) phospho-S6K/S6K levels were similar to initial CTR conditions (*Gba1* WT, 1.12 ± 0.20; *Gba1* KO, 0.76 ± 0.12; *Gba1* HET, 0.65 ± 0.11).

Analysis of phopho-S6K levels in a human patient-derived fibroblasts with homozygous and heterozygotic *GBA1* mutations, confirmed that *GBA1* deficient human fibroblasts had significantly reduced phopho-S6K levels under basal conditions (55% decrease in GBA deficient cells, *P <* 0.001) (Figure 2B) similar to *Gba1* deficient MEFs.

To verify that decreased phopho-S6K was due to lack of GCase activity we treated *Gba1* WT, KO and HET MEFs with imiglucerase (Cerezyme) which is used for therapeutic enzyme replacement of GCase in GD patients (2). Cerezyme (CER) treatment significantly increased GCase activity in *Gba1* KO and HET cells (*Gba1* WT 11.63 ± 1.69 nmol/h/mg; *Gba1* WT + CER 13.10 ± 2.37; *Gba1* KO 0.83 ± 0.18; *Gba1* KO + CER 3.04 ± 0.07 *P <* 0.001; *Gba1* HET 9.31 ± 0.77; *Gba1* HET + CER 11.2 ± 0.22; *P <* 0.05 (*n =* 3)). Cerezyme treatment significantly increased phospho-S6K levels in *Gba1* WT (34%, *P <* 0.05), *Gba1* KO (34%, *P <* 0.05) and *Gba1* HET (72%, *P <* 0.05) (Figure 2C), corroborating the direct relation between the loss of GCase activity and the decreased mTOR activity.

Alterations in lysosomal pH can lead to the changes in lysosomal function and in the lysosomal reformation process. The amount of acidic/functional lysosomes in the *Gba1* deficient cells was analysed using the Lyso ID probe. A significant decrease in acidic vesicles in both *Gba1* KO (67% ± 20.99, *P <* 0.01) and *Gba1* HET (40.9% ± 13.57, *P <* 0.001) was detected, when compared to *Gba1* WT MEF. This was not accompanied by alteration in lysosomal morphology or localization (data not shown). Treatment with Cerezyme resulted in a tendency to increase the acidic vesicles in the case of *Gba1* WT and *Gba1* KO (110% ± 16.96 and 82% ± 29.39) and *Gba1* HET (77% ± 20.93, *P <* 0.01) suggesting that GCase deficiency may be responsible for the lysosomal dysfunction (Figure 2D and E).

ALR impairment could be a consequence of the decreased lysosomal acidification, thus, we treated *Gba1* WT, KO and HET with 100nM of bafilomycin A1 (Baf A1) for 2h, which inhibits vacuolar H ^+^ ATPase (V-ATPase) and increases lysosomal pH. Upon treatment with Baf A1, *Gba1* WT cells had a trend to decrease phopho-S6K levels whereas, in both *Gba1* HET and *Gba1* KO cells Baf A1 treatment significantly decreased phospho-S6K, when compared to untreated controls (Fig. SI 2A). This suggests that lysosomal reformulation is affected by the alkalization of lysosomes.

### Rab 7 accumulates on lysosomes in GCase deficient cells

**Figure 3.**
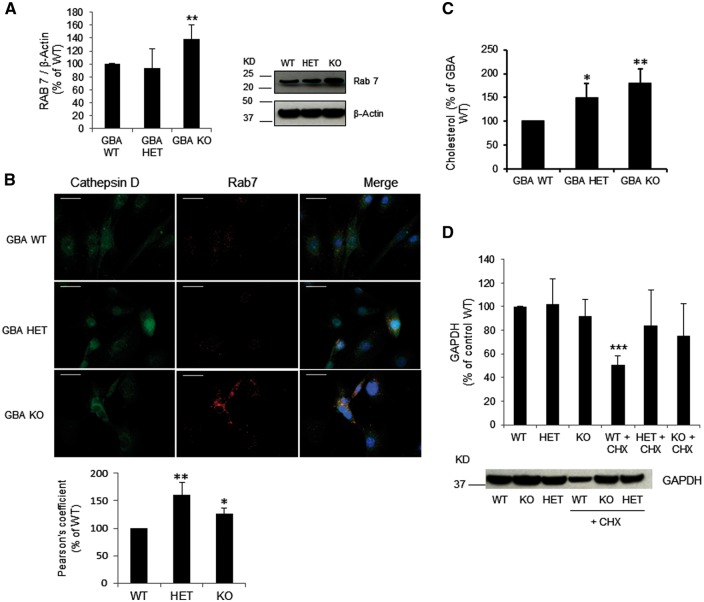
Rab7 accumulation, cholesterol accumulation and CMA impairment in GCase deficient cells. (**A**) Western blotting for Rab7 showed increased levels in *Gba1* KO cells compared to *Gba1* WT cells, while *Gba1* HET was unchanged (*n =* 3). (**B**) Co-localization of Rab7 (Red) with Cathepsin D (Green) and DAPI (blue); scale bar 25μm. *Gba1* HET and *Gba1* KO presented more punctate Rab7 staining and increased co-localization with cathepsin D. Graph represents co-localization quantification using Pearson’s Coefficient (*n =* 3). (**C**) Total cholesterol levels were increased in *Gba1* KO and *Gba1* HET compared to *Gba1* WT (*n =* 3). (**D**) GAPDH protein levels decreased in *Gba1* WT after 24h of protein synthesis inhibition with cycloheximide (CHX) (*n =* 3). No difference was observed in *Gba1* KO and *Gba1* HET MEF (*n =* 3). All data represent mean ± SD, *P < 0.05; **P < 0.01, ***P < 0.001 vs respective control.

The small GTP binding protein Rab7 has an important role in the maturation of endosomes and in the recycling of lysosomes by ALR (25,27). Rab7 levels were significantly increased in *Gba1* KO MEFs (38%, *P <* 0.01) (Figure 3A). Rab7 mRNA levels were unchanged (*Gba1* WT, 0.06 ± 0.02; *Gba1* KO, 0.06 ± 0.01; *Gba1* HET, 0.04 ± 0.01). We analysed whether Rab7 was accumulating on the lysosomes of GCase deficient cells by immunofluorescence (Figure 3B). Rab7 staining was more punctate in GCase deficient cells, and appeared to have a greater perinuclear localisation. The co-localisation between Rab7 and cathepsin D in *Gba1* KO and HET cells was increased compared to *Gba1* WT cells (*Gba1* KO 161% ± 22.66; *Gba1* HET 126% ± 11.04) (Figure 3B) suggesting slower lysosomal maturation/recycling (25,28). Increased cholesterol is a marker of immature/dysfunctional lysosomes (28). Total cholesterol levels in *Gba1* HET and KO cells were significantly increased (50%, *P <* 0.05 and 79%, *P <* 0.01, respectively), compared to *Gba1* WT cells (Figure 3C).

Lipidomic analysis also indicated that the cholesterol esters 15:1, 22:6 and 24:1 were significantly increased in *Gba1* KO cells by 4.8-fold (*P <* 0.001), 2.3-fold (*P <* 0.01), and 3.8-fold (*P <* 0.01), respectively, when compared to *Gba1* WT cells. No changes were seen in *Gba1* HET cells.

To analyse whether decreasing cholesterol levels in *Gba1* HET and KO restored ALR, we treated cells with Lovastatin (10μM for 48h), an HMG-CoA reductase enzyme inhibitor known to inhibit cholesterol production. Lovastatin decreased cholesterol levels by 43% in *Gba1* WT cells, 27% in *Gba1* KO and 26% in *Gba1* HET. This was the maximal dose and time before cell morphology and proliferation became affected. This treatment did not appear to restore ALR as phopho-S6K levels were not significantly changed in HET or KO following cholesterol decrease (Supplementary Material, Fig. SI 2B).

### Impairment of protein degradation via chaperone-mediated autophagy in GCase deficient MEFs

Impaired autophagy has also been linked to GCase deficiency, therefore we measured macroautophagy flux. LC3B-II protein level was analyzed in *Gba1* WT, KO and HET MEFs. There were no significant changes in LC3B-II protein levels under basal conditions in *Gba1* deficient cells compared to WT. Treatment with Baf A1 increased LC3B-II protein by a similar amount in all three cell lines, suggesting that there is not a significant effect on macroautophagy flux on these cells (Supplementary Material, Fig. SI 3A). Despite this finding, ATG16L, an essential protein for autophagy initiation, was significantly decreased in *Gba1* KO (49%, *P <* 0.001) and *Gba1* HET (23%, *P <* 0.001) compared to *Gba1* WT (Supplementary Material, Fig. SI 3B). mRNA levels of ATG16L were unchanged (*Gba1* WT, 3.24E-3 ± 3.80E-4; *Gba1* HET, 2.87E-3 ± 2.63E-4; *Gba1* KO, 3.36E-3 ± 1.23E-3). Immunofluorescence analysis showed that upon starvation ATG16L staining became more punctate and the number of puncta increased in *Gba1* HET and *Gba1* KO cells compared to *Gba1* WT. (Supplementary Material, Fig. SI 3C). These results may suggest a subtle dysregulation of macroautophagy.

We also assessed CMA activity. By measuring the turnover of the well-characterized CMA substrate, GAPDH, following protein synthesis inhibition with cycloheximide (CHX) for 24h, we detected that GAPDH protein levels were significantly decreased by 49.6% in *Gba1* WT MEF following CHX treatment, whereas GAPDH protein levels in *Gba1* HET and *Gba1* KO MEF were similar to non-treated cells (Figure 3D, *P <* 0.001). This suggests that in *Gba1* KO and HET, degradation via CMA is compromised. The protein levels of two key mediators of CMA, hsc70 and Lamp2a, were unchanged when compared to *Gba1* WT (Hsc70: *Gba1* HET, 108% ± 28.50; *Gba1* KO, 103% ± 27.70; Lamp2a: *Gba1* HET, 73% ± 15.00; *Gba1* KO, 78% ± 27.50).

### Knockdown of GCase in SH-SY5Y cells affects lysosomal content and lysosomal recycling

**Figure 4.**
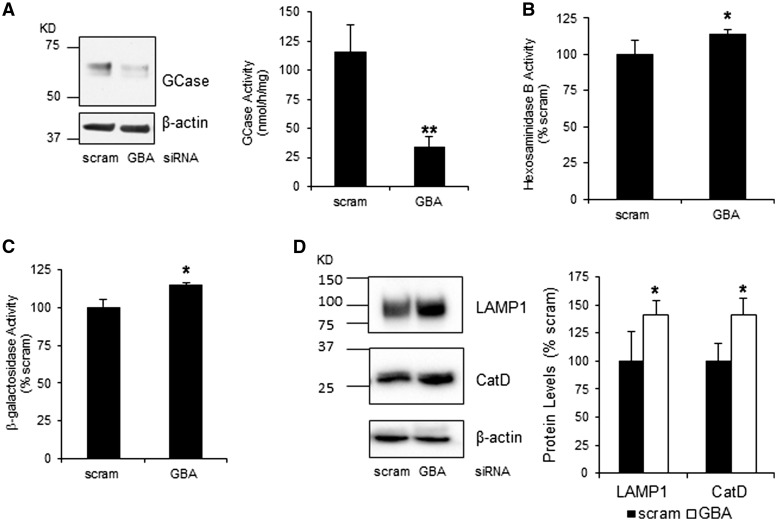
GCase knockdown in SH-SY5Y cells affects lysosomal content (**A**) GCase protein levels and activity are decreased in SH-SY5Y treated with GBA1 siRNA compared to scrambled control (*n =* 5). Enzyme activity of (**B**) β-hexosaminidase and (**C**) β-galactosidase were significantly increased in *GBA1* siRNA-treated cells (*n =* 5). (**D**) Protein levels of mature cathepsin D and LAMP1 were also increased in *GBA1* siRNA KD cells (*n =* 5). All data represent mean ± S.E.M. * *P <* 0.05, ** *P <* 0.01.

To assess ALR and the turnover of α-synuclein we knocked-down (KD) GCase by siRNA in parental SH-SY5Y cells and SH-SY5Y over expressing human α-synuclein. GCase protein expression was decreased by 56 ± 6% following 10 days of KD with *GBA1* siRNA, when compared to cells treated with scrambled siRNA (Figure 4A). This resulted in a significant 70% decrease in GCase activity (Figure 4A). The activities of the lysosomal enzymes β-hexosaminidase and β-galactosidase and the protein expression levels of mature cathepsin D protein and LAMP1 were all significantly increased in *GBA1* KD cells (Figure 4B–D).

**Figure 5.**
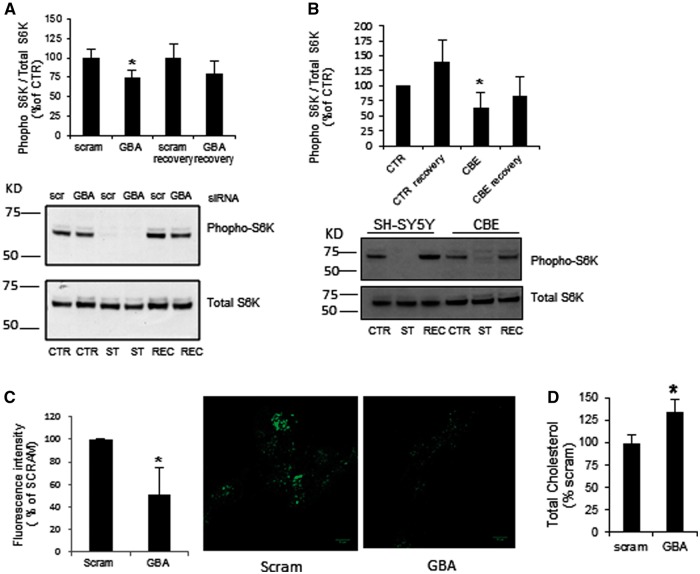
Impairment of ALR in GCase deficient SH-SY5Y cells. (**A**) Under basal conditions (CTR) phospho-S6K was significantly decreased in GCase KD cells. Following starvation (ST) and recovery (REC), S6K phosphorylation recovered although was less in GCase KD cells (*n =* 5). (**B**) CBE-treated cells presented decreased phopho-S6K levels under basal conditions and the recovery was less than in untreated cells (*n =* 3), (**C**) Lyso ID fluorescence measurements detected a significant decrease in acidic functional lysosomes in GCase KD cells (*n =* 3). Scale bar 10µm. (**D**) Total cholesterol levels were increased in *GBA1* siRNA-treated cells compared to scramble treated cells (*n =* 7). All data represent mean ± SD, * *P <* 0.05.

Under basal conditions (CTR), phospho-S6K was significantly decreased by 25.6% in *GBA1* KD cells (Figure 5A; *P <* 0.05, *n =* 5). Starvation-induced autophagy for 2 h (ST) inhibited mTOR, resulting in loss of S6K phosphorylation. Culturing of these cells with media containing serum for 1 h (REC) recovered S6K phosphorylation, but was less in GCase KD cells (Figure 5A). To confirm that this was due to GCase deficiency, S6K phosphorylation was assessed in SH-SY5Y treated with the GCase inhibitor conduritol-β-epoxide (CBE; 10 μM). This concentration of CBE inhibited GCase activity by 92.0 ± 0.0% after 10 days of treatment (replenished every 3 days). Phospho-S6K was significantly decreased by 37% under basal (CTR) conditions in CBE-treated cells, and did not recover as much as untreated cells (SH-SY5Y) once starvation-induced autophagy had been removed for 1 h (REC; Figure 5B).

The acidic/functional lysosomes were quantified in this model using the Lyso-ID probe. GCase KD SH-SY5Y cells presented a decreased number of functional/acidic lysosomes compared to control (Figure 5C; 51% ± 23.58, *P <* 0.05). Changes in lysosomal morphology and cellular localization were not detected (data not shown).

Analyses of GCase KD cells indicated that the 70% decrease in GCase activity did not result in an increase in the substrate glucosylceramide or other sphingolipids such as ceramide and sphingomyelin (data not shown). Total cholesterol levels were increased by 135 ± 13% (*P <* 0.05, *n =* 7) in GCase KD cells (Figure 5D).

### Knockdown of GCase in SH-SY5Y cells inhibits macroautophagy flux and increases α-synuclein protein levels

**Figure 6.**
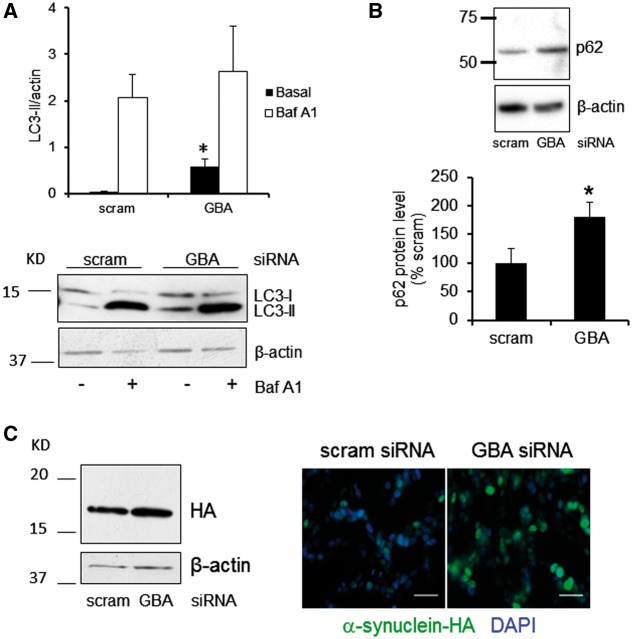
Knockdown of GCase in SH-SY5Y cells inhibits macroautophagy flux and increases α-synuclein protein levels. (**A**) Western blot for LC3B-II levels, under basal conditions, showed a significant increase in LC3B-II levels in *GBA1* siRNA-treated cells. Both scramble and siRNA cells treated with Baf A1 increased LC3B-II levels to a similar extent (*n =* 4). (**B**) Autophagic protein p62 was significantly increased in *GBA1* siRNA-treated cells compared to scramble-treated cells (*n =* 6). (**C**) Western blot and immunohistochemistry showed increase α-synuclein protein levels in *GBA1* siRNA-treated SH-SY5Y expressing α-synuclein tagged with hemagglutinin (HA) (*n =* 5). Scale bar 25μm. All data represent mean ± SD * *P <* 0.05 vs. scram.

Macroautophagy flux was measured by western blot analysis of LC3B-II levels under basal conditions, or in the presence of 100nM Baf A1 (Figure 6A). Under basal conditions, LC3B-II levels were significantly increased in *GBA1* siRNA-treated cells (*P <* 0.05). Treatment with Baf A1 increased LC3B-II/β-actin ratio to a similar level in scrambled or siRNA treated cells (scram siRNA LC3B-II/β-actin, 2.07 ± 0.50; *GBA1* siRNA, 2.64 ± 0.96, *n =* 4), indicating that the clearance of autophagosomes was impaired in *GBA1*-siRNA treated cells. Protein levels of the autophagic protein p62 were also significantly increased in *GBA1* siRNA-treated cells (Figure 6B; *P <* 0.05), further confirming an inhibition of macroautophagy flux in these cells. Protein levels of CMA proteins Lamp2a and Hsc70 were unchanged (data not shown).

GCase KD in SH-SY5Y cells over expressing exogenous WT α-synuclein tagged with hemagglutinin (HA) resulted in a significant increase in α-synuclein protein levels (Figure 6C; scram siRNA, 0.65 ± 0.18; *GBA1* siRNA, 1.01 ± 0.06; *P =* 0.05, *n =* 5). HA-tagged α-synuclein was also found to be increased in these cells by immunofluorescence following treatment with *GBA1* siRNA (Figure 6C).

### Impaired lysosomal recycling and α-synuclein accumulation in GCase deficient neurons

**Figure 7.**
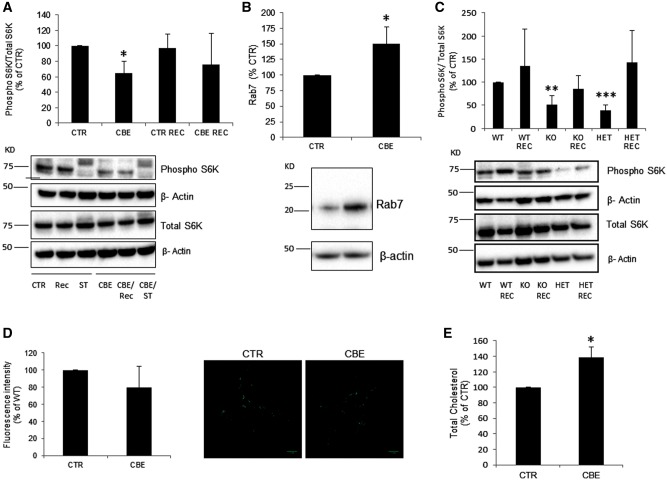
Impairment of ALR in GCase deficient cortical neurons. (**A**) Cortical neurons treated with CBE (10 days) presented decreased basal levels of phopho-S6K compared to untreated (*n =* 3). Levels of phopho-S6K following recovery were consistently lower in CBE-treated neurons. (**B**) Neurons treated with CBE for 10 days had increased Rab7 protein levels compared to control (CTR) cells as measured by western blot. (**C**) *Gba1* KO and HET cortical neurons presented decreased phopho-S6K levels compared to *Gba1* WT under basal conditions (*n =* 4). Phopho-S6K levels increased upon recovery (REC) in all cases. (**D**) Lyso ID fluorescence measurements detected a decrease in acidic functional lysosomes in CBE-treated neurons (*n =* 4) but was not significant. (E) Total cholesterol levels were increased in neurons treated with CBE compared to untreated neurons (*n =* 3). Results were expressed as % of control. All data represent mean ± S.E.M, * *P <* 0.05; **P <  0.01; ***P <  0.001 vs. respective control.

We treated WT neurons with CBE and analysed phopho-S6K levels to detect defects in the ALR in GCase deficient cortical neurons. After 10 days of treatment, CBE inhibited GCase activity by 97.0 ± 0.0%. Phospho-S6K was significantly decreased by 35% under basal (CTR) conditions in CBE-treated neurons. Once starvation-induced autophagy had been removed for 1 h (REC), phospho-S6K levels increased but levels were still lower than CTR (Figure 7A). Rab7 protein levels were also significantly increased in CBE-treated neurons (Figure 7B). To verify if ALR was also compromised in *Gba1* KO mice neurons, we compared phospho-S6K levels in *Gba1* KO and *Gba1* HET to WT *Gba1* cortical neurons (Figure 7C). We found that the basal levels of phospho-S6K were lower in *Gba1* KO and *Gba1* HET neurons compared to *Gba1* WT neurons (*Gba1* KO 51.0% ± 19.55, *P <* 0.01; *Gba1* HET 39% ± 11.25, *P <* 0.001, *n =* 4).

Acidic/functional lysosomes were quantified in GCase deficient cortical neurons using the Lyso-ID probe. There was a trend for a decrease in the number of functional lysosomes in GCase deficient neurons compared to control (Figure 7D; 80.0% ± 24.5, *n =* 4). Changes in lysosomal morphology and localization were not detected (data not shown).

Similar to MEFs and SH-SY5Y, loss of GCase activity resulted in an increase in total cholesterol levels by 138 ± 23% (*P <* 0.05, *n =* 3) in CBE-treated neurons compared to untreated neurons (Figure 7E).

**Figure 8.**
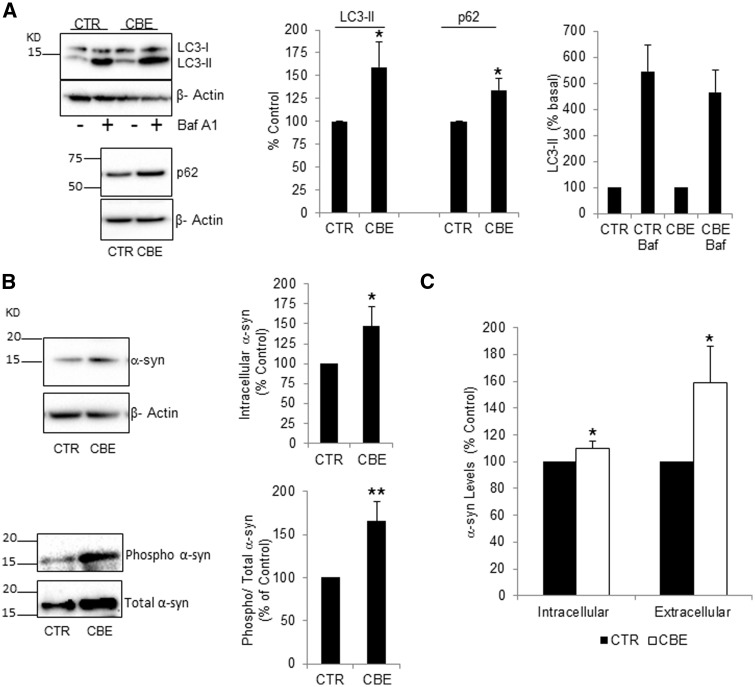
GCase deficiency inhibits macroautophagy flux and increases α-synuclein protein levels in cortical neurons. (**A**) LC3B-II and p62 were measured in neurons under basal conditions, or following treatment with 100 nM Baf A1 for 6 h. LC3B-II and p62 levels were significantly increased in CBE-treated neurons (*n =* 7). (**B**) Intracellular total α-synuclein (*n =* 5) and phospho Ser129 α-synuclein (*n =* 6) were measured in neurons treated with CBE. (**C**) α-synuclein levels were measured in cell lysates (intracellular) and their respective conditioned cell culture media (extracellular) by ELISA. Data are from four independent neuron preps. All data represent mean ± S.E.M, * *P <* 0.05; ** *P <* 0.01 vs. respective control.

Under basal conditions, LC3B-II levels were significantly increased by 159% in CBE-treated neurons (Figure 8A). Treatment with Baf A1 increased LC3B-II to a similar amount in CTR and CBE-treated cells suggesting the increase in LC3B-II under basal conditions was due to inhibition of macroautophagy flux (Figure 8A). In support of this, the autophagic cargo protein p62 was also significantly increased by 134% (Figure 8A). Levels of the CMA proteins hsc70 and Lamp2a were unaffected (Supplementary Material, Fig. SI 4A, B).

Inhibition of macroautophagy significantly increased intracellular total monomeric α-synuclein levels by 147% as measured by western blotting (Figure 8B). Phosphorylation of α-synuclein at Ser129 has been reported in α-synuclein aggregates and Lewy bodies. Western blotting indicated that phospho-Ser129 α-synuclein was significantly increased by 165% in CBE–treated neurons (Figure 8B). Furthermore, probing of intracellular lysates with an antibody that recognizes all types of amyloid oligomers (e.g. α-synuclein, huntingtin or Aβ42) indicated that 4/7 independent CBE-treated neuron cultures exhibited higher molecular weight protein species (>50 kDa) that were not detected in control neurons (Supplementary Material, Fig. SI 4C). These bands were increased by co-treating neurons with 25 nM bafilomycin A1 for 48 h suggesting these amyloid oligomeric species were due to inhibition of lysosomal activity.

Measurement of monomeric α-synuclein in cell lysates by ELISA confirmed that intracellular levels were significantly increased in GCase-deficient neurons (Figure 8C). The release of α-synuclein into cell media from these same neurons was also significantly increased by 159%, when compared to untreated neurons (Figure 8C). The amount of α-synuclein detected in media (CTR, 0.70 ± 0.10 ng/mg protein; CBE, 1.11± 0.29 ng/mg protein) is a small fraction compared to intracellular α-synuclein levels (CTR, 1109 ± 146 ng/mg protein; CBE, 1237 ± 163 ng/mg protein).

## Discussion

**Figure 9.**
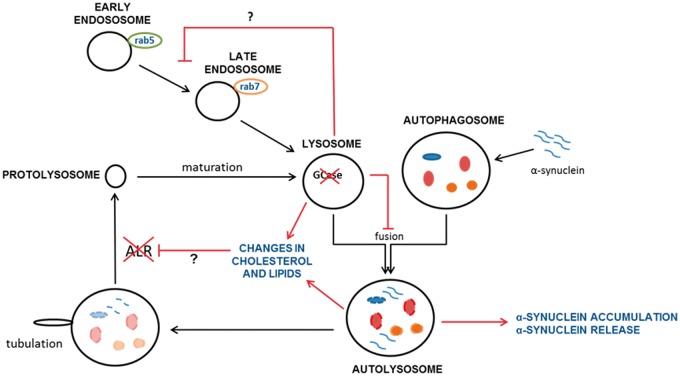
Loss of lysosomal GCase causes impairment of ALR and maturation of endosomes, perhaps due to changes in lipids and/or cholesterol. Over time this leads to a decrease in the functional pool of lysosomes in the cell impairing autophagy and resulting in the cellular accumulation of α-synuclein and increased release of α-synuclein contributing to PD pathology.

We report that the inhibition of autophagy following GCase deficiency is due to loss of lysosomal biogenesis via ALR and the maturation of endosomes. These events occurred in several cellular models of heterozygous and homozygous GCase deficiency suggesting that this is a mechanism by which pathogenic α-synuclein species could accumulate in PD (Figure 9).

ALR is a vital process for the cell to restore its pool of functional lysosomes. This process is initiated upon attenuation of autophagy due to reactivation of mTOR, which was measured by the increased phosphorylation of S6K (25). In all cases of GCase deficiency, phospho-S6K was decreased suggesting mTOR was less active in these cells. Upon starvation followed by recovery, phospho-S6K levels in GCase deficient cells remained lower than in controls. Therefore over time, repeated cycles of autophagy followed by ALR are going to result in less functional lysosomes in GCase deficient cells as evidenced by the decreased number of acidified lysosomes and expression and activity of other lysosomal enzymes. Neurons differentiated from iPSC with homozygote or heterozygote *GBA1* mutations have shown lysosomal dysfunction and impaired autophagy, with lysosomal cell content either increasing (29) or decreasing (15,30). Notably, we report that ALR impairment and loss of acidic lysosomes could be reversed by treating cells with recombinant GCase (Cerezyme).

The GTPase Rab7 has an important role in endosome-lysosome maturation (27,28,31) and is also required to dissociate from autolysosomes for ALR to proceed (25). In GCase deficient cells, we detected both an accumulation of Rab7 and increased co-localization with cathepsin D suggesting Rab7 does not dissociate from the autolysosomes as quickly as in control cells. Inhibition of ALR has been shown to increase Rab7 protein levels and have increased association with autolysosomes (32). Furthermore, these same changes in Rab7 and the accumulation of cholesterol in late endosomes have also been associated with impaired maturation of lysosomes (28,33). Dysregulation of Rab7 has been linked with mutations in the Leucine-rich repeat kinase 2 (*LRRK2*) gene, which cause autosomal dominant PD (34–36), suggesting that impaired maturation of lysosomes and ALR could be a common mechanism in PD pathogenesis.

Specific lipids have been implicated in the maturation of endosomes and ALR (32,33). Increased cholesterol also impairs the maturation of late endosomes to lysosomes (33,37). Therefore, changes in glucosylceramide or other lipid and sterol species as a result of GCase deficiency may contribute to the decrease in lysosomal maturation and ALR. ALR inhibition has also been reported to occur in fibroblasts with saposin c deficiency (38). Saposin c is a cofactor for GCase and saposin c deficiency in these cells resulted in accumulation of glucosylceramide (38). Inhibition of GCase with CBE, or administration of exogenous glucosylceramide, increases cholesterol, inhibits endosomal degradation and alkalizes endosomes/lysosomes (37,39). Corroborating our data, two studies showed cholesterol accumulation in patient derived skin fibroblasts bearing GCase mutations (40,41).

As cholesterol accumulated in our models we studied whether decreasing cholesterol levels, using Lovastatin, a drug widely used to treat hypercholesterolaemia, would restore ALR (42). We did not detect a restoration of ALR in *Gba1* KO and HET cells when cholesterol levels were lowered, although the maximum non-toxic dose of Lovastatin was unable to restore levels back to normal. Statins such as Lovastatin have wide range of effects in the cells, not only decreasing cholesterol levels but affecting other pathways including activation of AKT in neurons. (43). AKT is known to be upstream of mTOR. While total cellular cholesterol levels are reduced by Lovastatin, we cannot exclude the possibility that cholesterol is still increased in endosomes and lysosomes. Therefore, we do not exclude the possible importance of cholesterol/lipid dysregulation in ALR impairment following GCase deficiency.

Substantial evidence suggests a role for autophagy impairment and lysosomal depletion in PD and ageing (20). This not only results in increased intracellular α-synuclein levels, but also increased release and transmission of α-synuclein to other cells (44,45). Our neuronal GCase deficiency models exhibited inhibition of macroautophagy, increased intracellular total and phosphorylated (S129) monomeric α-synuclein levels and release of α-synuclein, co-incident with impaired ALR. In line with our findings, a recent report showed increased release of α-synuclein in iPSC-derived dopamine neurons from the patients with PD with heterozygous *GBA1* mutations (29). The amount of α-synuclein in condition media was a small fraction compared to intracellular levels (< 1%). We measured release in to media for 72 h, so it is likely that some α-synuclein had been degraded or taken back up by neurons during this time.

A proportion of CBE-treated neurons (4 out of 7 cultures) were also positive for amyloid-like deposits. The antibody cannot distinguish whether these oligomers contain α-synuclein or other amyloidgenic proteins such as β-amyloid or huntingtin. Since phospho Ser129 α-synuclein, which is typically found in α-synuclein aggregates, is also increased in these cells, and α-synuclein aggregates have previously been reported in other GCase deficient models (14,17,19), it is likely that these amyloid deposits contain α-synuclein. However, further work is required to identify the composition of these putative oligomers.

We did not find impairment in macroautophagy flux in our *Gba1* mutant MEFs. In previous studies no evidence of macroautophagy inhibition was found in GD fibroblasts (38,46). However, macroautophagy has been shown to be impaired in mixed cultures of neurons and astrocytes from the same mouse model (19).

Loss of ALR appeared to be similar in our heterozygote or homozygote *GBA1* models. Autophagy inhibition and α-synuclein accumulation have previously been reported to be similar in iPSC-derived neurons from GD patients and the patients with PD with heterozygous *GBA1* mutations (15). Genetic studies also support these observations, as both GD and heterozygotic *GBA1* gene carriers present a similar risk of developing PD (47).

We propose that loss of functional lysosomal GCase leads to impairment of lysosomal recycling and endosome maturation, possibly due to alterations in lipid and/or cholesterol homeostasis. Over time, repeated cycles of autophagy followed by impaired ALR cause a decrease in the pool of functional lysosomes and thus autophagy deficiency, diminishing the cellular quality control mechanisms which results in α-synuclein accumulation and increased α-synuclein release, contributing to PD pathology. Since recombinant GCase can reverse ALR impairment, we anticipate that strategies to restore GCase activity in the brains of both sporadic patients with PD and those with *GBA1* mutations will improve autophagy lysosomal pathway, preventing the accumulation of α-synuclein and spread of pathology.

## Materials and Methods

### Ethical approval

Dermal fibroblasts derived from controls (*n =* 2) or patients with GD or PD were generated following local ethical approval from the Hampstead Research Ethics Committee: reference number 10/H0720/21. All individuals provided written informed consent.

The Gba1 knockout mouse model colony was covered by project licence 70/7685 issued by the United Kingdom Home Office. All animal procedures were carried out in accordance with the United Kingdom Animals (Scientific Procedures) Act of 1986 (Schedule 1). All efforts were made to reduce the number of animals by following the 3R's.

### Cell culture lines

SH-SY5Y cells and SH-SY5Y cells expressing exogenous wild-type α-synuclein containing a hemagglutinin tag (HA) (22) were cultured in 1:1 DMEM/F12 (Invitrogen, Carlsbad, CA, USA) supplemented with 10% FBS, non-essential amino acids, 1 mM sodium pyruvate and penicillin-streptomycin.

Dermal fibroblasts derived from patients with GD and PD were used: one N370S homozygotic mutation, one N370S/wt heterozygotic mutation, one WT/null allele, one L444P/wt. Fibroblasts were cultured in DMEM supplemented with 10% FBS, 1 mM sodium pyruvate and penicillin-streptomycin (48).

Heterozygotic *Gba1* transgenic mice in which a loxp-neo-loxp (lnl) cassette was inserted into exon 8 of one *Gba1* allele in all cell types (26) were used to generate mouse embryonic fibroblasts (MEFs) as follows. Skin from the torso of *Gba1^+/+ ^*(*Gba1* WT), *Gba1^-/-^*(*Gba1* KO), and *Gba1^+/-^* (*Gba1* HET) day 15 embryos was dissected, minced and cultured in DMEM supplemented with 10% FBS, 1mM sodium pyruvate and penicillin-streptomycin (all from Invitrogen) and an antibiotic-antimycotic mixture (Sigma, ST Louis, MO, USA). After 48h adherent cells were passaged in to fresh media supplemented as above but without antibiotic-antimycotic mixture. MEFs were continuously passaged every three days until obtaining selection of immortalized cells (∼15 passages) (49).

WT mice and heterozygotic *Gba1* transgenic mice (26) were used to generate primary cortical neurons as follows. Cortexes from day 15 embryos were dissected, meninges removed, homogenized by passing through a 23G needle and centrifuged at 1000rpm for 5 min. Cell pellets were resuspended in neurobasal media (Invitrogen) supplemented with B27 (Invitrogen), glutamax (Sigma) and antimycotic/antibiotic solution (Sigma) and seeded onto poly-ornithine coated plates (Sigma). Neurons were left in culture for no longer than 15 days.

#### Silencing of *GBA1* and inhibition of GCase activity

SH-SY5Y cells (1.8 x 10^5^ cells/ml) were transfected with 25 nM *GBA1* siRNA (Dharmacon, Lafayette, CO, USA; sense: GGAUGUGCCUCUU- ACCAUCUU; antisense: GAUGGUAAG AGGCA CAUCCUU) or 25 nM scrambled control #1 (Ambion, Foster City, CA, USA) for 3 days. SH-SY5Y were then passaged and transiently transfected as above with siRNA. This was repeated on days 6 and 9. Cells were harvested on day 10.

Conduritol-β-epoxide (CBE; Universal Biologicals, Cambridge, UK) was used to inhibit GCase activity in SH-SY5Y and cortical neurons. Cells were continuously treated with 10μM of CBE every 3 days for 10 days.

#### Cerezyme treatment

*Gba1* WT, HET and KO MEFs were treated with 12ug/ml of imiglucerase (Cerezyme; Genzyme, MA, USA) for 3 days, with fresh media containing Cerezyme replaced daily.

#### Starvation and recovery assay

SH-SY5Y, MEFs and neurons were plated in 6 well plates. Cells were treated with either complete media or media lacking FBS (starvation media). After 2h of starvation, cells were either harvested, or for recovery studies, media were replaced with complete media and cells harvested after 1h incubation.

#### Western blotting

Cells were harvested with trypsin, washed in phosphate buffered saline (PBS), and lysed in 1% (v/v) Triton X-100 in PBS supplemented with protease and phosphatase inhibitors. Nuclei and cell debris were removed by centrifugation at 17, 000*g*. For intracellular detection of total monomeric α-synuclein levels in neurons, cell pellets were lysed in 0.1% SDS, 150 mM NaCl, 10mM Tris, pH7.5 supplemented with RQ1 DNase (Promega) and protease and phosphatase inhibitors and incubated at 37 °C for 1 h. For detection of oligomeric species, cells were lysed with RIPA buffer on ice (150 mM NaCl, 1% (v/v) NP-40, 1% (w/v) sodium deoxycholate, 50 mM Tris, pH 8.0) supplemented with protease inhibitors. Debris was removed by centrifugation as above. Protein (5–30 μg) was separated on 4–12% or 12% Bis-Tris NuPAGE gels (Invitrogen) and transferred to a Hybond P membrane (GE Healthcare, Little Chalfont, UK). Blots were incubated with antibodies against Cathepsin D (CTD-19, 1:2000, abcam, Cambridge, UK), LAMP1 (1:1000 Abcam), LAMP2a (1:1000 Abcam) Hsc70 (EP1531Y, 1:500 Abcam), GCase (2E2, 1:2000 Calbiochem, Billerica, MA, USA), HA (HA.11, 1:2000, Covance, Princeton, NJ, USA), LC3B (2775, 1:1000, Cell Signaling, Danvers, MA, USA), LC3B (1:500, Abcam), p62 (610833,1:500, BD Biosciences, Franklin Lakes, NJ, U.S), SQSTM1/p62 (1:1000, Abcam), Atg16L (D605, 1:500, Cell Signaling), phopho - p70S6Kinase (T389, 1:500, Cell Signaling), p70S6Kinase (1:500, Cell Signaling), Rab7 (EPR7589, 1:1000 Abcam), α-synuclein (4D6, 1:1000; Abcam), phospho (S129) α-synuclein (1:1000, Abcam), amyloid oligomers (A11, 1:1000, Abcam) or β-actin (1:2000, Abcam). Bands were detected with respective horse radish peroxidase-linked secondary antibodies (Dako, Glostrup, Denmark) and enhance chemiluminescence (Pierce, Waltham, MA, USA) or Luminata Forte (Merck, Millipore). Density of bands was determined using ImageJ software (NIH) or Image Lab software (BioRad).

#### GAPDH turnover

GAPDH turnover was assessed using cycloheximide (25µg/ml) assay. Samples were harvested 24h after treatment. Samples were loaded according to cell number calculated using a fluorescent DAPI assay of DNA content using a plate reader (excitation, 360nm; emission 460 nm). Western Blot was performed as described previously using anti-GAPDH primary antibody (ab8245, 1:1000 Abcam).

### Lysosomal enzyme assays

Cell lysates were prepared as above, sonicated and GCase activity determined in samples (20 μg protein) by hydrolysis of 5 mM 4-methylumbelliferyl-β-D-glucopyransoside in McIIvaine buffer (pH 5.4) in the presence of 22 mM sodium taurocholate at 37 °C for 1 h. The reaction was stopped by the addition of 0.25M glycine (pH 10.4) and 4-methylumbelliferone fluorescence measured at excitation 360 nm, emission 460 nm.

β-hexosaminidase and β-galactosidase were assayed in lysates (20μg protein) using the fluorogenic substrates 4-methylumbelliferyl-N-acetyl-glucosaminide (2mM) or 4-methylumbelliferyl-β-D-galactopyransoside (0.25 mM), respectively, in sodium citrate buffer (pH 4.2) at 37 °C for 30 min (50,51). The reaction was stopped by the addition of 0.25M glycine (pH 10.4) and fluorescence measured as above.

### Lysosomal function: Lyso-ID green detection kit

Acidic vesicles were detected in live cells using the Lyso-ID Green detection kit (Enzo Lifesciences, Farmingdale, NY, USA). Cells were seeded in 96-well plates or 12 well plates (90% confluent) and incubated with 4 μl/ml Lyso-ID in assay buffer supplemented with 2% (v/v) FCS at 37 °C for 30 min. Cells were washed with assay buffer and fluorescence measured on a plate reader at excitation 488 nm, emission 520 nm. Following reading, the buffer was aspirated, cells lysed overnight in 0.25M NaOH and protein concentration measured. Cell fluorescence was expressed as fluorescent units/mg protein.

For live cell imaging, cells were seeded on glass bottom dishes (ibidi, Germany) and incubated with 1μl/ml Lyso-ID in HBSS for 15 min. Cells were then washed with HBSS and imaged in HBSS.

### Immunofluorescence

Cells were fixed in 3.7% paraformaldehyde (pH 7.4), permeabilised with methanol for 10min at -20^°^C, blocked in 2% (v/v) goat serum in PBS with 0.1% (v/v) Triton X-100 for 1h at RT, and then incubated with HA antibody (HA.1, 1:1000, Covance) at 37 °C, or ATG16L (D605, 1:100, Cell Signaling), cathepsin D (CTD-19, 1:100, Abcam) and Rab7(EPR7589, 1:100 Abcam) antibodies overnight at 4^°^C. Following washing of cover slips with PBS, cells were incubated with anti-mouse or anti-rabbit alexa fluor (Invitrogen) for 1h at 37 °C, washed with PBS and coverslips mounted in citifluor containing DAPI.

### Image acquisition and processing

Imaging was performed in a Nikon Eclipse Ti-E inverted microscope system using a 60x/1.4 oil objective and a Hamamatsu ORCA- Flash 4.0 camara.

Images were acquired using NIS Elements AR software. Lyso ID signal intensity was measured using Image J software. Rab7, Cathepsin D co-localization was measured using Image J plug-in JACoP (52).

#### Measurement of α-synuclein by ELISA

Neurons were cultured in poly-ornithine-coated 12-well plates for 14 days. Neurons were treated with 10 μM CBE on days 4, 9 and 11. Conditioned media were removed from the neurons on day 14 and debris/floating cells removed by centrifugation. Neurons were harvested with trypsinisation and cell pellets and their respective conditioned media frozen. Total cell pellets were lysed in RIPA buffer and protein content calculated using a BCA protein assay kit (ThermoScientific). The amount of intracellular α-synuclein and α-synuclein released in to the media was measured by ELISA (Sensolyte; AnaSpec) as per manufacturer’s instructions. The data were expressed as pg α-synuclein/mg protein from at least three separate wells per prep. The percent increase of α-synuclein in CBE-treated neurons was then calculated from four independent neuronal cultures.

#### Lipidomic analysis

Cellular lipids were extracted in methanol/chloroform (2:1, v/v). The lipids were analyzed by liquid chromatography-mass spectrometry (LC-MS) using a Thermo Exactive Orbitrap mass spectrometer (Thermo Scientific, Hemel Hempstead, UK), equipped with a heated electrospray ionization probe and coupled to a Thermo Accela 1250 UHPLC system. The samples were analyzed in positive and negative ion modes over m/z 200-2000. Injections (1 µl) were made onto a Thermo Hypersil Gold C18 column (1.9 μm; 2.1 mm × 100 mm). Mobile phase A consisted of water containing 10 mM ammonium formate and 0.1% (v/v) formic acid. Mobile phase B consisted of 90:10 isopropanol/acetonitrile containing 10 mM ammonium formate and 0.1% (v/v) formic acid. The initial conditions for the analysis were 65%A/35%B. The percentage of mobile phase B was increased to 100% over 10 min and held for 7 min before re-equilibration with the starting conditions for 4 min. The LC-MS data were processed with Progenesis QI software v2.0 (Non-Linear Dynamics, Newcastle upon Tyne, UK) and searched against LIPID MAPS (www.lipidmaps.org/) and the Human Metabolome Database (http://www.hmdb.ca/) for identification. The mean abundance of identified molecular species of glucosylceramide within the study groups was calculated from the normalised positive ion data sets.

#### Cholesterol measurements

Total cholesterol levels were analysed using the Amplex Red Cholesterol Assay kit from Invitrogen. Manufacturer instructions were followed and cholesterol levels were expressed in µg of total cholesterol/mg of total protein.

#### Lovastatin treatments

Cells were treated with 10µM of Lovastatin (Sigma) for 48H and cholesterol was measured as described before.

### Statistical analysis

Statistical analyses were performed by Student’s t test. Values with *P <* 0.05 were considered significant.

## Supplementary Material

Supplementary Material is available at *HMG* online.

Supplementary Data
