# Supplementary material for: Autophagic lysosome reformation dysfunction in glucocerebrosidase deficient cells: relevance to Parkinson disease
Source: Hum Mol Genet. 2016 Jul 4;25(16):3432–45. doi: 10.1093/hmg/ddw185 (PMC5179940; doi:10.1093/hmg/ddw185)
Supplement: Supplementary Data [file supp_25_16_3432__index.html]

Autophagic lysosome reformation dysfunction in glucocerebrosidase deficient cells: relevance to Parkinson disease — Autophagic lysosome reformation dysfunction in glucocerebrosidase deficient cells: relevance to Parkinson disease — Supplementary Data 

# Autophagic lysosome reformation dysfunction in glucocerebrosidase deficient cells: relevance to Parkinson disease

## Supplementary Data

files

- Supplementary Data - zip file
