# Supplementary material for: Autophagic lysosome reformation dysfunction in glucocerebrosidase deficient cells: relevance to Parkinson disease
Source: Hum Mol Genet. 2016 Jul 4;25(16):3432–45. doi: 10.1093/hmg/ddw185 (PMC5179940; doi:10.1093/hmg/ddw185)
Supplement: Supplementary Data [file supp_ddw185_suppl_data.zip › HMG-2016-D-00181_SI_Magalhaes.docx]

**Supplementary Information**

**Figure Legends**

**Figure SI 1.** Altered glucosylceramide sphingolipid content in GCase deficient MEF cells. Lipids were extracted from *Gba1* WT, *Gba1* KO and *Gba1* HET MEFs (~1 x 10^6^ cells) and (A) total glucosylceramide species and (B) individual species detected by liquid chromatography-mass spectrometry. Values are % of total ion content (TIC) and are expressed as the mean±SEM (n=3 separate cultures).

**Figure SI 2.** (A) GCase deficiency in *Gba1* HET and *Gba1* KO MEFs causes changes in lysosomal acidity. *Gba1* WT, HET and KO (n=4), were treated with 100uM of Bafilomycin A1 (Baf A1) for 2h and phopho-S6K levels were detected. Results were expressed in % of respective control. Treatment with Baf A1 showed a decrease in phopho-S6K levels in *Gba1* KO and *Gba11* HET compared to respective CTR (no BAF A1), while *Gba1* WT showed a tendency to have decreased levels of phopho-S6K that did not reach significance. (B) *Gba1* WT, HET and KO (n=4), were treated with 10uM of Lovastatin for 48h and phopho-S6K levels were detected. Results were expressed in % of respective control. Treatment with lovastatin showed an increase in phopho-S6K basal levels in *Gba1 WT MEFs,* but no significantly changes in phopho-S6K in *Gba1* KO and *Gba1* HET. Upon starvation-recovery protocol no changes were detected in either *Gba1* WT, KO or HET. All data represent mean±SD, *p<0.05; **p<0.01, ***p<0.001.

**Figure SI 3.** GCase deficiency causes autophagy impairment. (A) Western blot for LC3B-II showed no significant change in LC3B-II levels in *Gba1* KO and HET MEF cells compared to *Gba1* WT upon basal conditions or following incubation with Baf A1(100nM for 6h) (n=6). (B) ATG16L protein levels in *Gba1* KO and *Gba1* HET were significantly decreased compared to *Gba1* WT levels (n=4). (C) ATG16L immunofluorescence in *Gba1* WT, *Gba1* HET and *Gba1* KO (n=3) under basal conditions and after 2h starvation. Images showed that, under basal conditions, *GBA1* WT cells had more ATG16L staining than *Gba1* HET or *Gba1* KO. After starvation *Gba1* HET and *Gba1* KO cells presented a greater accumulation of punctuated ATG16L staining compared to *Gba1*WT cells (ATG16L, red; DAPI, Blue; scale bar 25 μm). All data represent mean ± SD, ***p<0.001.

**Figure SI 4.** CMA proteins are unaffected in neurons treated with CBE. Neurons treated with CBE for 10 days did not exhibit changes in the CMA proteins hsc70 (A) or LAMP2A (B). Data are the mean ±SEM (n=5). (C) Neurons were lysed in RIPA buffer and immunoblotted with an antibody that detects amyloid oligomers. Higher molecular weight bands (>50 kDa, arrows), were detected in 4/7 neuronal cultures treated with CBE for 10 days. Treatment of CBE treated neurons with 25 nM bafilomycin A1 for the last 48 hours increased the proportion of higher molecular species suggesting these are a result of impaired lysosomal function (right panel).
