# Supplementary figures and images for: Autophagic lysosome reformation dysfunction in glucocerebrosidase deficient cells: relevance to Parkinson disease
Source: Hum Mol Genet. 2016 Jul 4;25(16):3432–45. doi: 10.1093/hmg/ddw185 (PMC5179940; doi:10.1093/hmg/ddw185)

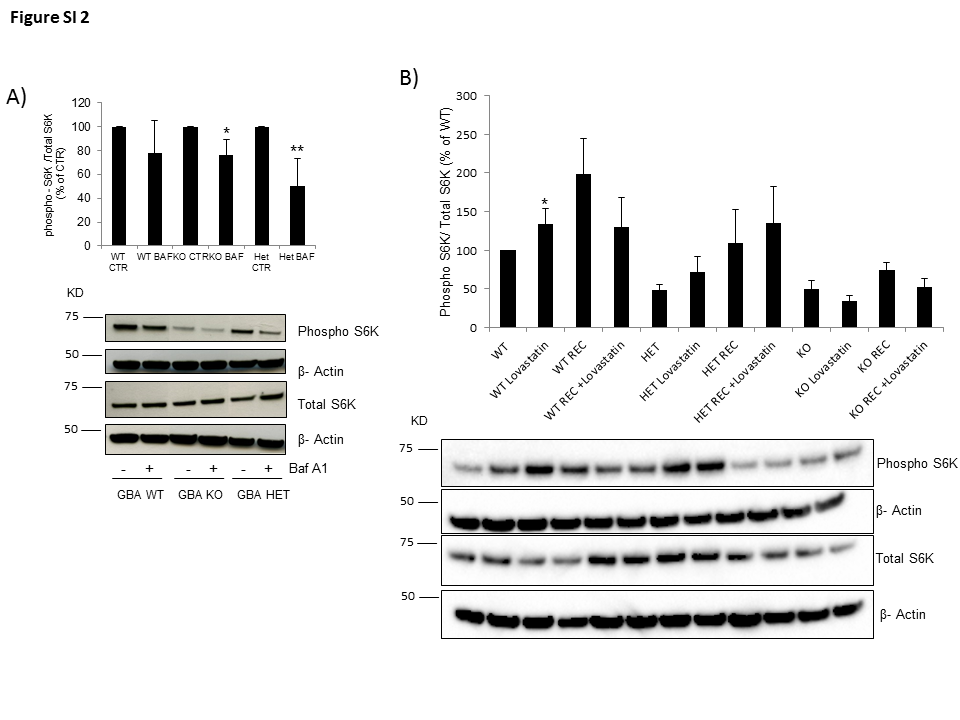

Supplement: Supplementary Data [file supp_ddw185_suppl_data.zip › SI_FIG_2.tif]

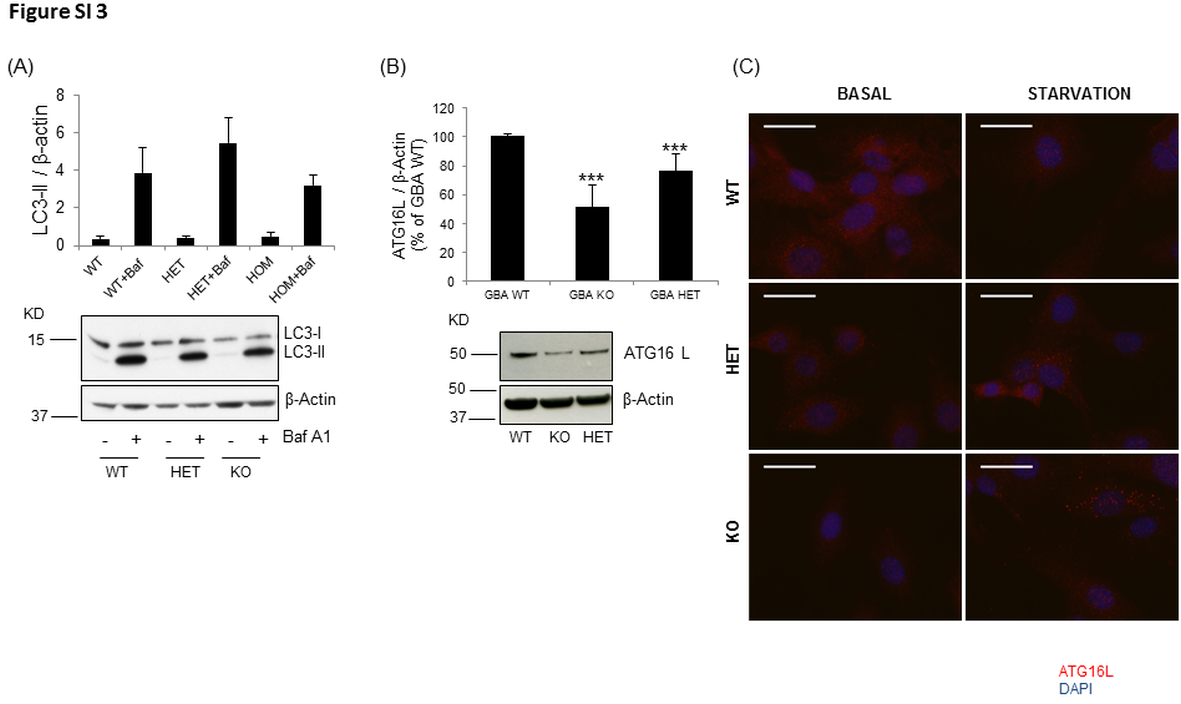

Supplement: Supplementary Data [file supp_ddw185_suppl_data.zip › SI_FIG_3.tif]

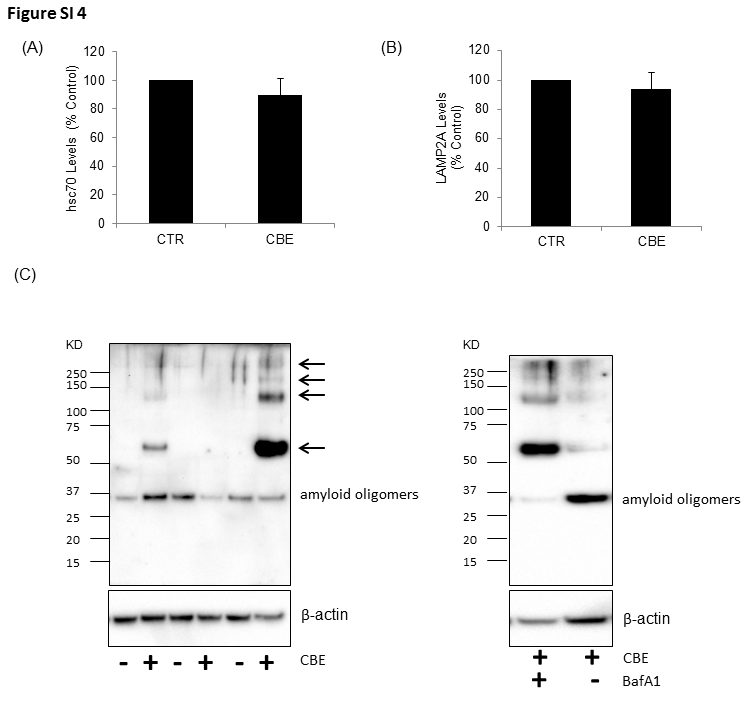

Supplement: Supplementary Data [file supp_ddw185_suppl_data.zip › SI_FIG_4.tif]

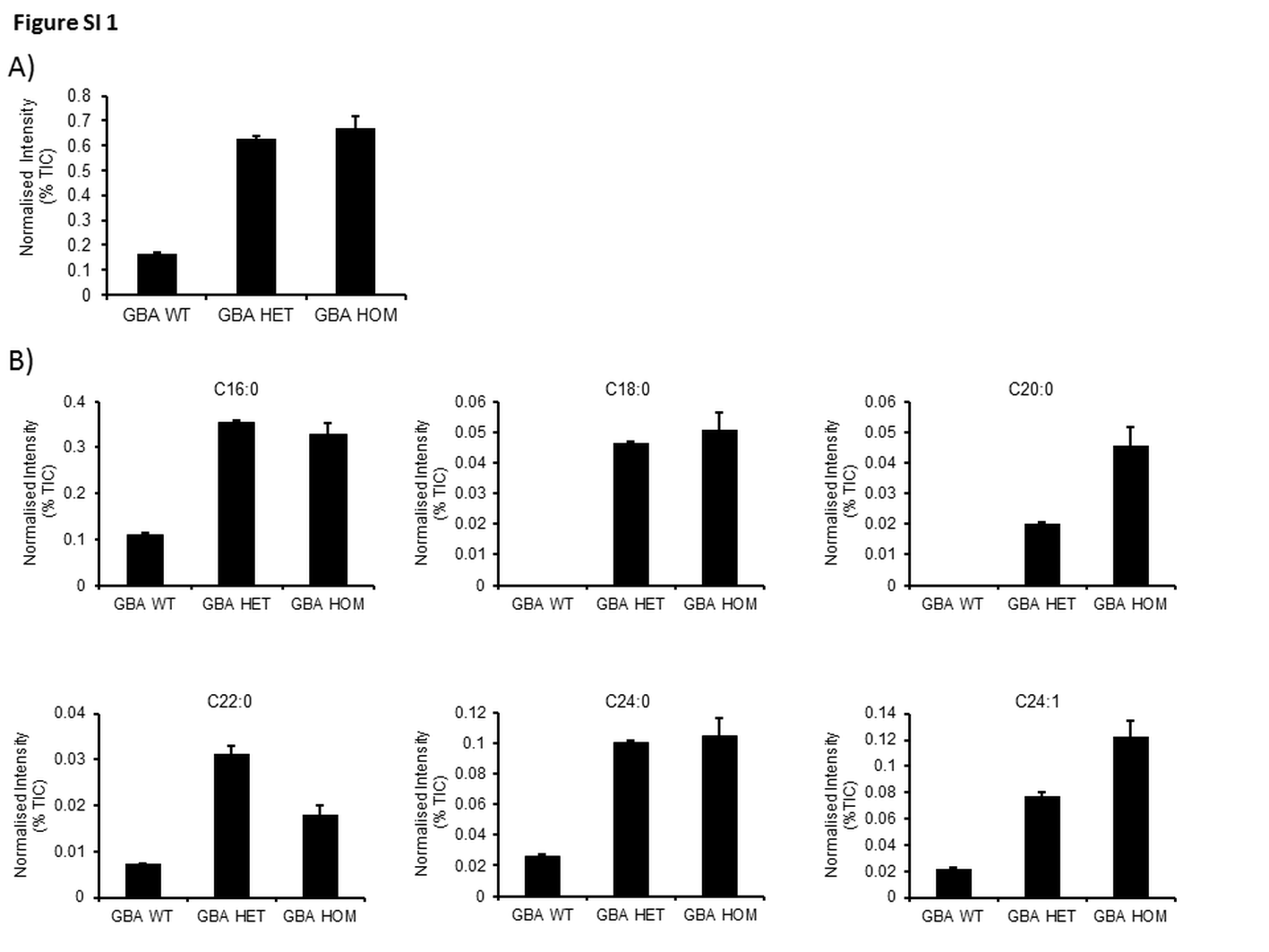

Supplement: Supplementary Data [file supp_ddw185_suppl_data.zip › SI FIG_1.tif]
